# The combination of balloon-assisted antegrade transvenous obliteration and transjugular intrahepatic portosystemic shunt for the management of cardiofundal varices hemorrhage

**DOI:** 10.1097/MEG.0000000000001705

**Published:** 2020-01-23

**Authors:** Jiacheng Liu, Chongtu Yang, Songjiang Huang, Chen Zhou, Qin Shi, Kun Qian, Songlin Song, Bin Xiong

**Affiliations:** a Department of Radiology, Union Hospital, Tongji Medical College, Huazhong University of Science and Technology; b Hubei Province Key Laboratory of Molecular Imaging, Wuhan, China

**Keywords:** balloon-occluded retrograde transvenous obliteration, cardiofundal varices, gastric varices, portal vein thrombosis, transjugular intrahepatic portosystemic shunt

## Abstract

**Objectives:**

In this study, we propose a modified balloon-occluded retrograde transvenous obliteration (BRTO) strategy – balloon-assisted antegrade transvenous obliteration (BAATO), and explore the feasibility, efficacy and safety of BAATO combined with transjugular intrahepatic portosystemic shunt (TIPS) in the treatment of cardiofundal varices (GOV2 or IGV1) hemorrhage.

**Materials and methods:**

In this retrospective cohort study, 15 patients with cardiofundal varices hemorrhage who received BAATO combined with TIPS procedures, from August 2017 to September 2019 in our center, were enrolled. They consisted of seven patients with GOV2 and eight patients with IGV1. The clinical efficacy and safety of BAATO + TIPS procedures were assessed by comparing the clinical symptoms, laboratory and imaging examinations before and after treatment.

**Results:**

The technical success rate of BAATO + TIPS procedure was 100%. After the procedure, clinical symptoms were improved and complete regression of gastric varices (GVs) was observed in all patients, besides, the control efficiency of ascites and PVT which were 77.8 and 87.5%, respectively. No patient died or had a rebleeding during the follow up, but grade II hepatic encephalopathy (HE) occurred in two patients (13.3%) and shunt dysfunction was discovered in one patient (6.7%).

**Conclusion:**

For the treatment of GVs, the new technique BAATO is feasible, safe and effective, and it may be a more convenient and economical method than conventional BRTO. In addition, the combination of BAATO and TIPS may play a positive role in achieving hemostasis and improving the complications of portal hypertension such as ascites and PVT.

## Introduction

Gastric varices (GVs) are present in around 20% of patients with portal hypertension [1]. According to Sarin’s classification [2], GV is divided into four types including gastroesophageal varices type 1 (GOV1), GOV type 2 (GOV2), isolated GV type 1 (IGV1) and isolated GV type 2 (IGV2), among which GOV2 and IGV1 are commonly referred to as ‘cardiofundal varices’. Currently, transjugular intrahepatic portosystemic shunt (TIPS) is the treatment of choice in the control of bleeding from cardiofundal varices, and TIPS ± balloon-occluded retrograde transvenous obliteration (BRTO) or BRTO ± TIPS have been listed as a first-line treatment in secondary prevention [3,4].

BRTO technique was first developed and named by Kanagawa *et al.* [5]. During this procedure, the occlusion balloon catheter is advanced into the GVs through gastrorenal shunt (GRS), and sclerosant was injected into GVs with the occlusion of GRS [6,7]. Before the sclerosant injection, small collateral veins should be embolized by coils generally to avoid the sclerosant move into systemic circulation through them [8]. In addition, the occlusion balloon catheter should be placed for 5 h or longer under balloon occlusion in order to maximize the residence time of sclerosant in GVs and minimize the occurrence of complications [9,10].

Therefore, we raised a modified procedure: balloon-assisted antegrade transvenous obliteration (BAATO). In brief, the retrograde occlusion balloon catheter is used to occlude the GRS, followed by antegrade trans-TIPS catheter injecting cyanoacrylate rather than sclerosant. The distribution of cyanoacrylate in GVs could be controlled by adjusting the balloon size for the blood flow velocity varies as the balloon size changes. The advantages of this procedure are as follows: (1) It is a one-stop management because the GVs embolization can be performed simultaneously with TIPS; (2) The blood flow velocity could be controlled by adjusting the balloon size, so that cyanoacrylate can spread and embolize GVs all over; (3) Extra embolization of small collateral veins could be avoided, and the balloon catheter can be withdrawn immediately after the procedure instead of being placed for a long time, which makes it a handy technique that also reduces time cost and medical resources.

Here we reported 15 patients with cardiofundal varices (GOV2 or IGV1) who had undergone BAATO + TIPS procedure, and investigated the efficacy and safety of this new combined treatment besides the convenience of this procedure.

## Materials and methods

This retrospective cohort study was conducted according to the Helsinki Declaration, and written informed consent was obtained for every procedure from all patients. The protocol was approved by the Wuhan Union Hospital Biomedical Research Ethics Committee.

### Patients

This study included 15 consecutive patients with cardiofundal varices hemorrhage from August 2017 to September 2019 in our center. The inclusion criteria were as follows: (1) Upper gastrointestinal bleeding, (2) cardiofundal varices confirmed by endoscopic examination, (3) contrast-enhanced computed tomography (CT) of the portal system revealed the presence of GRS, (4) treatment with BAATO + TIPS procedures. The exclusion criteria were as follows: (1) severe liver, kidney or heart failure, (2)uncontrollable systemic infection or septicemia, (3) polycystic liver, (4) extensive primary or metastatic hepatic malignancy and (5) uncontrolled coagulopathy [11].

**Fig. 1:**
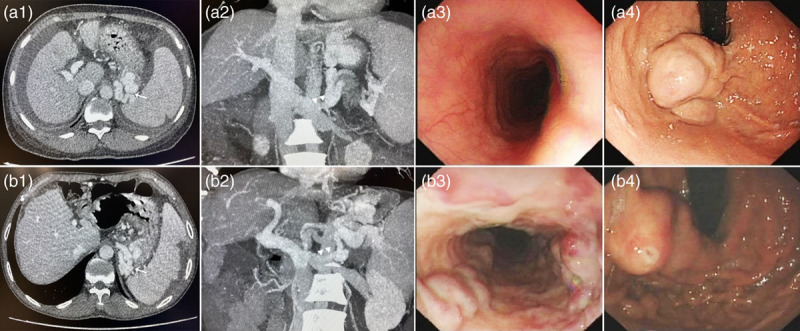
. (a) A 54-year-old female bleeding from IGV1. (a1 and a2) Enhanced CT showing solitary GV bolus (a1, white arrow) and GRS (a2, white triangles); (a3 and a4): endoscopic examination showing only GV but no esophageal varices (EV). (b) A 57-year-old male bleeding from GOV2. (b1 and b2) Enhanced CT showing GV bolus (b1, white arrow) and GRS (b2, white triangles); (b3 and b4) endoscopic examination showing both EV and GV. CT, computed tomography.

Before the procedure, all patients reached a stable state of hemodynamics through vasoactive drugs, volume resuscitation, balloon tamponade or endoscopic therapies. And they routinely received laboratory tests such as liver and kidney function, blood routine and coagulation function. Endoscopic examination was used to classify GVs (according to the Sarin endoscopic classification). And contrast-enhanced CT or MRI was used to plan out the BAATO + TIPS procedure (Fig. 1) [12].

### BAATO + TIPS procedure

**Fig. 2.**
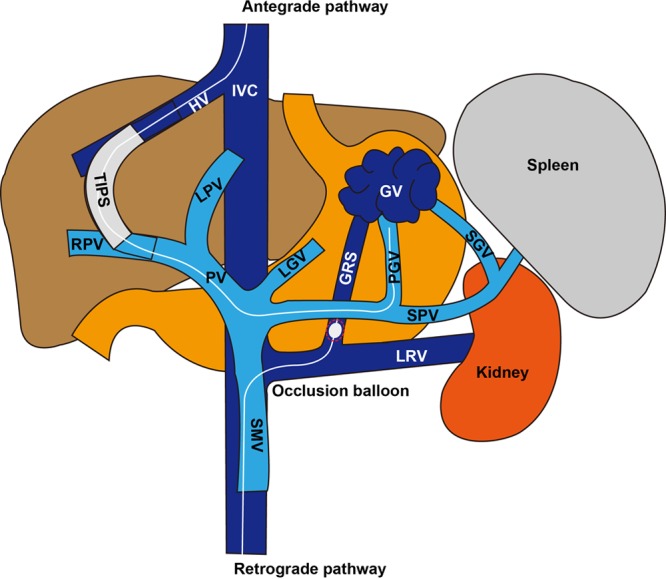
The schematic diagram of BAATO + TIPS procedure, which demonstrates the constructions of antegrade pathway (portal circulation) and retrograde pathway (systemic circulation). TIPS was placed through the antegrade pathway and cyanoacrylate injection was performed through the cooperation of antegrade catheter and antegrade balloon. GRS, gastrorenal shunt; GV, gastric varices; HV, hepatic vein; IVC, inferior vena cava; LGV, left gastric vein; LPV, left portal vein; LRV, left renal vein; PGV, posterior gastric vein; PV, portal vein; RPV, right portal vein; SGV, short gastric vein; SMV, superior mesenteric vein; SPV, splenic vein; TIPS, transjugular intrahepatic portosystemic shunt.

(1) Construction of retrograde pathway: a sheath introducer was introduced into the left renal vein via the right femoral vein under local anesthesia; next, a occlusion balloon catheter (Fogarty; Edward, Indiana, USA) was advanced into the GRS; then portography was performed to visualize GVs in detail and check feeding veins, draining veins and small collateral veins.
(2) Construction of the antegrade pathway: the catheterization of the hepatic vein was performed through the right internal jugular vein with a transjugular liver access set (RUPS-100; Cook Inc., USA); once the shunt between hepatic vein and portal vein was successfully established, the catheter was then advanced into the main portal vein, superior mesenteric vein or splenic vein, and portography was then performed again to visualize GVs and portal pressure gradient (PPG) value was obtained subsequently.
(3) Cyanoacrylate injection: the trans-TIPS catheter was advanced into the GV, then the suspension of N-butyl-cyanoacrylate (Compant, Beijing, China) and lipiodol was injected. The ratio of cyanoacrylate to lipiodol was 1:1 ~ 1:3, which varied according to the size of GVs and blood flow velocity, however, generally, 1:2 was adopted. while cyanoacrylate injection was performed, the size of retrograde occlusion balloon was slightly reduced to guarantee a small quantity of blood through GRS to carry cyanoacrylate alongside the vessel to fill GVs all over. During the procedure, the operator and assistants should pay attention not to let cyanoacrylate move into systemic circulation by adjusting the balloon size.
(4) TIPS placement: after the intrahepatic tract was dilated with a 6 mm balloon catheter (Bard Inc., Karlsruhe, Germany), an 8 mm bare stent (E-Luminexx; Bard Inc.) combined with an expanded polytetrafluoroethylene covered stent (Fluency; Bard Inc.) were implanted between the hepatic vein and the portal vein, post-stenting venogram and PPG measurement were obtained again; finally, the balloon catheter was relaxed and withdrawn (Fig. 2) [13,14].

### Follow-up

Laboratory and imaging examinations were performed at 1, 3, 6 and 12 months after the procedure, followed by every 6 months [15]. The occurrence of hepatic encephalopathy (HE), variceal rebleeding, survival and shunt dysfunction were monitored during the follow-up. Laboratory examinations include: total bilirubin (TB), albumin (ALB), alanine aminotransferase, aspartate aminotransferase, creatinine, blood urea nitrogen, prothrombin time, international normalized ratio and platelet count, etc. Endoscopy and contrast-enhanced CT were performed to confirm the complete regression of GV, and evaluate the condition of ascites and portal vein thrombosis (PVT).

We maintained frequent contact with the patients by telephone and a brief interview was conducted to determine their clinical course after BAATO + TIPS procedures. HE was evaluated and graded based on the West Haven criteria [16].

### Statistical analysis

Statistical analysis was performed with SPSS 22.0 (IBM Corporation) and GraphPad Prism 8 was used for figures. Continuous variables were presented as mean ± SD and compared using Student’s *t*-test. While categorical variables were presented as percentage ratio and compared by corrected Chi-squared test or Fisher’s exact test.

## Results

### Patient characteristics

This study consisted of 15 patients, including 11 (73.3%) males and four (26.7%) females, and they were averagely aged 56.0 ± 6.3 years old. Among them, nine (60.0%) patients were diagnosed with HBV-induced cirrhosis, three (20.0%) were HCV-induced cirrhosis and the other three (20.0%) were alcoholic cirrhosis. And seven (46.7%) patients were classified as GOV2, eight (53.3%) were IGV1, all of whom were complicated with GRS. According to Child-Pugh classification, two (13.3%) patients were rated A, 11 (73.3%) were rated B and two (13.3%) were rated C. The average Child-Pugh score, model for end-stage liver disease (MELD) score and MELD-Na score were 7.7 ± 1.3, 12.8 ± 1.6 and 14.4 ± 2.5, respectively. Patient characteristics are summarized in Table 1.

**Table 1.**
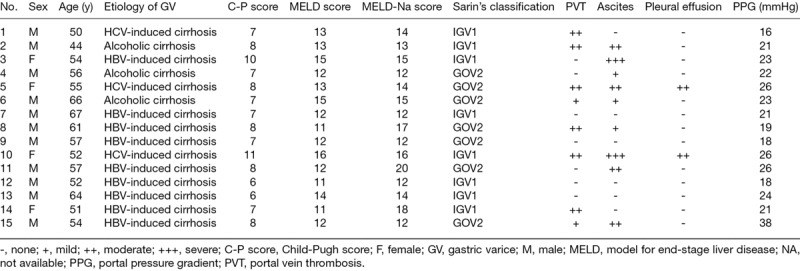
Baseline characteristics of all the 15 patients

BAATO + TIPS procedure was completed in all patients with a technical success rate of 100%. The GVs of 5 (33.3%) patients were formed from posterior gastric vein, seven (46.7%) were from short gastric vein and three (20.0%) were from both the posterior gastric vein and the short gastric vein. The mean diameter of the largest variceal was 8.4 ± 1.5 mm. The average PPG value decreased from 22.8 ± 5.2 mmHg to 10.5 ± 3.7 mmHg (*P* < 0.001), and the decrease ratio all exceeded 30% of the baseline value.

### Efficacy and safety

After the procedure, the symptoms of hematemesis, melena of all patients disappeared and no fatal complications occurred, such as renal failure, pulmonary embolism or liver failure.

**Fig. 3.**
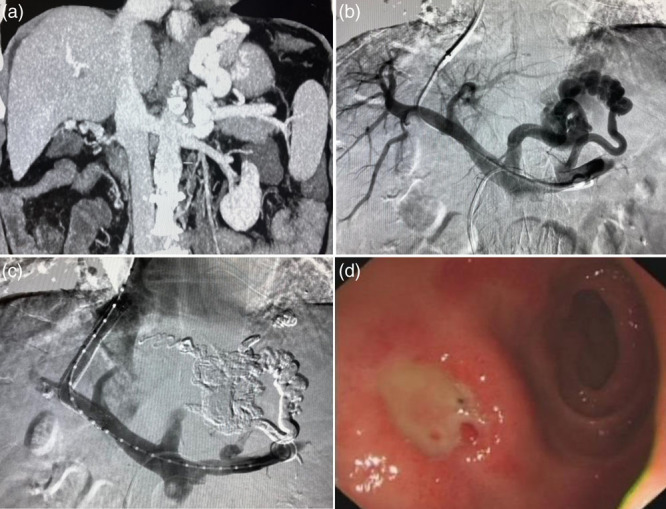
A 67-year-old male bleeding from IGV1. (a) Before the BAATO + TIPS procedure, enhanced CT showed solitary GVs bolus and GRS. (b) During the procedure, the portography showed GVs feeded of posterior gastric vein and short gastric vein; (c) post-stenting venogram showed that cyanoacrylate spreaded and embolized GVs all over. (d) After the BAATO + TIPS procedure, endoscopic examination showed the complete regression of GVs. CT, computed tomography; GRS, gastrorenal shunt.

One month after the procedure, complete regression of GV was observed in all patients (Fig. 3), and the regression rate was 100%. Before the procedure, nine patients (60.0%) were complicated with ascites, and only two (15.5%) of them were left 1 month after the procedure. And the cases of PVT dropped from eight (53.3%) to one (6.7%) after the procedure. The condition of both ascites and PVT showed a tendency of improvement, the control efficiency of ascites and PVT were 77.8 and 87.5%, respectively.

The median follow-up time of these 15 patients was 6 (range: 2–27) months. During the follow-up, no patient died and no rebleeding was observed. Grade II HE occurred in two patients, which makes the cumulative rate of HE 13.3%. Shunt dysfunction occurred in one patient and was restored after balloon dilatation.

### Laboratory parameters

**Fig. 4.**
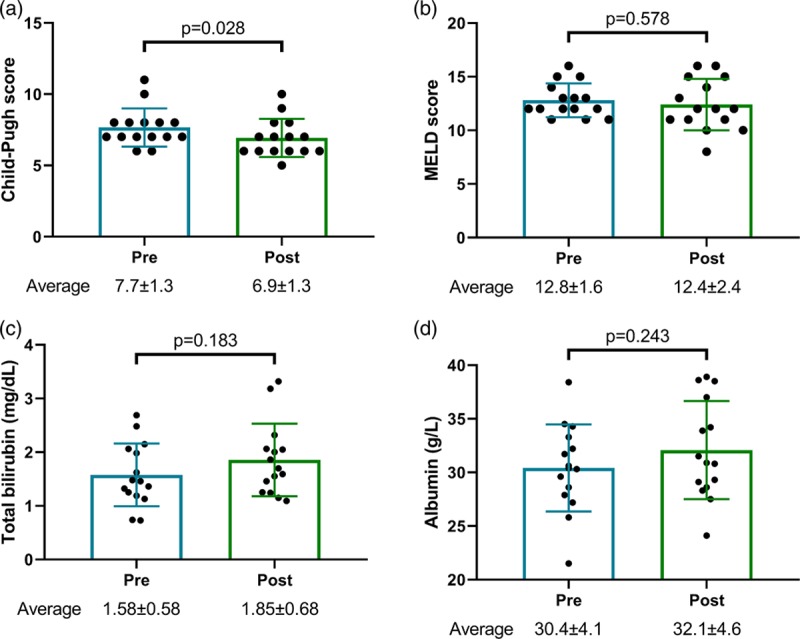
The Child-Pugh score (a), MELD score (b), total bilirubin (c) and albumin (d) of all patients before and one month after the procedure. MELD, model for end-stage liver disease.

Demonstrated in Fig. 4 was the Child-Pugh score, MELD score, TB and ALB of all patients before and 1 month after the procedure. Specifically, the Child-Pugh score dropped from 7.3 ± 1.3 to 6.7 ± 1.3 (*P* = 0.024), which marked a trend for the better. However, no significant difference was found concerning MELD score, TB and ALB before and after the procedure.

## Discussion

The treatments for GVs may vary depending on a variety of factors such as the patient’s liver function, severity of coexisting esophageal varices, the presence of GRS, and size/angle of GRS [17]. And the management options mainly consist of two aspects: therapies that reduce portal pressure (β-blockers, TIPS and shunt surgery) and procedures that directly target GVs (endoscopic therapy and BRTO) [4]. Conventional endoscopic treatments include endoscopic variceal ligation (EVL) and endoscopic injection sclerotherapy. EVL, however, could only be operated in small GVs, and the band may slip after a few days, leaving ulcers on the surface of blood vessel, which would induce a great risk of rebleeding [18]. Moreover, conventional endoscopic treatment has been proved to act poorly in controlling the bleeding rate and rebleeding rate [19]. Therefore BRTO, on the other hand, is a better choice for treatment of cardiofundal varices (GOV2 or IGV1) associated with GRS [3].

BRTO is a technique that sclerosant is injected through the catheter when the balloon completely blocks the shunt. And the balloon is usually retained for a few hours after the injection of sclerosant in order to ensure enough time of the sclerosant in GV and minimize the side effects. Since BRTO was proposed, the placement of an indwelling balloon for hours has always been the core procedure of this technology [5,9,20]. But high level of nursing qualification is needed while waiting for the withdrawal of the balloon catheter, which also requires secondary procedure to withdraw of the balloon catheter, and so it is rather complex and a waste of medical resources. Previously, some have proposed modified BRTO techniques such as coil-assisted retrograde transvenous obliteration (CARTO) [21] and vascular plug-assisted retrograde transvenous obliteration (PARTO) [22], which utilized coils or vascular plug embolization to replace the indwelling occlusion balloon catheter [23]. Although the retention of the balloon could be avoided, limitations are still obvious. In CARTO procedure, for example, it requires a large number of coils to be able to replace the balloon as a barrier, which means it is expensive, complicated and time-consuming. In terms of PARTO procedure, it is affected by the size of GRS due to limitations on size of the vascular plug (22 mm), and it may be impossible to reach 100% blockage because of the mesh material of vascular plug [24].

Thus, we propose a new modified method – BAATO, GVs embolization is performed by the cooperation of antegrade trans-TIPS catheter and retrograde occlusion balloon catheter. Cyanoacrylate is injected into GVs through the antegrade catheter and the distribution of cyanoacrylate could be controlled by adjusting the balloon size. While cyanoacrylate injection was performed by the operator, the balloon size can be slightly reduced by the assistant to guarantee a small quantity of blood through GRS to carry cyanoacrylate alongside the vessel to fill GVs all over, once the cyanoacrylate found to move into systemic circulation, the injection should stop and balloon dilation should be performed immediately. After N-butyl-cyanoacrylate enters the GVs, it solidifies and adheres to the vessel wall in a very short time [25]. Therefore, it is unnecessary to perform extra embolization of small collateral veins using coils because the cyanoacrylate is unlikely to move into the systemic circulation through those small collateral veins. On the other hand, this modified method could also avoid the indwelling balloon for hours, which will help reduce the burden of nursing and save medical resources. Through the size adjustion of the balloon, we could ensure that the cyanoacrylate is distributed completely in the GVs. As for the ratio of cyanoacrylate to lipiodol, it depends on the size of GV and blood flow velocity. The GV being larger and the blood flow being faster, the ratio should be bigger correspondingly. In this study, all the patients have received BAATO treatment, and their GVs have reached complete regression and no complication was observed due to cyanoacrylate entering the systemic circulation. In addition, the cumulative rate of variceal rebleeding was 0% during the follow-up, which all together confirms the feasibility and safety of BAATO treatment.

All 15 patients in our study were treated with BAATO + TIPS procedure. We think that, on the one hand, trans-TIPS BAATO could reach GVs through already applied TIPS to avoid additional percutaneous approach through the liver, which makes it a one-stop treatment [26]. On the other hand, after BAATO procedure, the resultant expected increase in portal pressure and attendant increased risk of new/worsening ascites, aggravation of GV, and formation of ectopic varices can be ameliorated by the simultaneous placement of TIPS [27]. Indeed after the procedure, the PPG of these 15 patients decreased significantly, the bleeding stopped immediately, and the GVs all underwent a complete regression. Encouragingly, the situation of ascites and PVT was alleviated, and the Child-Pugh score also decreased.

Previous studies have shown that although BRTO alone could be positive in GVs bleeding, the portal pressure still increases due to the occlusion of GRS [28], which aggravates the ascites, worsens PVT and deteriorates liver function [29,30]. Consequently, it is necessary to reduce the portal pressure through TIPS while blocking GVs. As shown in our results, the combination of BAATO and TIPS may be helpful in avoiding potential risks of new/worsening ascites and aggravation of PVT caused by BRTO alone.

Last but not least, we must admit our limitation that as a retrospective study, the sample size was small and the follow-up time was not long enough. But we hold expectations that it will be more convincing when we keep following up on these patients and gather more patients in the future. Besides, for patients with large GRS diameter, it remains a problem to choose the suitable diameter of occlusion balloon. Although for patients with small GRS diameter, if with enough cyanoacrylate adhered to the vascular wall, balloon-assisted occlusion may not be necessary, and the cyanoacrylate could be simply injected through the antegrade catheter, which is similar to the endoscopic cyanoacrylate injection for GOV1.

In conclusion, for the treatment of GVs, the new technique BAATO is feasible, safe and effective, and it may be a more convenient and economical method than conventional BRTO. In addition, the combination of BAATO and TIPS may play a positive role in achieving hemostasis and improving the complications of portal hypertension such as ascites and PVT.

## Acknowledgements

We would like to thank all colleagues for helping us during the current study.

This work was funded by the National Natural Science Foundation of China (81873917).

## Conflicts of interest

There are no conflicts of interest.
